# Investigating the impact of the diseases of despair in Appalachia

**DOI:** 10.13023/jah.0102.02

**Published:** 2019-07-06

**Authors:** Michael Meit, Megan Heffernan, Erin Tanenbaum

**Affiliations:** NORC at the University of Chicago, meit-michael@norc.org; NORC at the University of Chicago, heffernan-megan@norc.org; NORC at the University of Chicago, Tanenbaum-Erin@norc.org

**Keywords:** opioids, overdose, mortality, Appalachia

## Abstract

**Introduction:**

Appalachia is one of the regions most significantly impacted by the opioid crisis. This study investigated mortality due to diseases of despair within the Appalachian Region, with an additional focus on deaths attributable to opioid overdose.

**Methods:**

Diseases of despair include: alcohol, prescription drug and illegal drug overdose, suicide, and alcoholic liver disease/cirrhosis of the liver. Mortality data from the National Center for Health Statistics (NCHS) National Vital Statistics System (NVSS) Multiple Cause of Death database were analyzed for this study, focusing on individuals aged 15–64.

**Results:**

Over the past two decades, the mortality rate due to diseases of despair has been increasing across the United States, but the gap has widened between the Appalachian Region and the rest of the nation. In 2017, the combined diseases of despair mortality rate was 45% higher in the Appalachian Region than the non-Appalachian United States. When looking at just overdose mortality, this disparity grows to 65% higher in the Appalachian Region. Within the Appalachian region disparities are most notable in the Central and North Central Appalachian subregions, among males, and among individuals age 45 to 54.

**Discussion:**

These findings document the scale and scope of the problem in Appalachia and highlight the need for additional research and discussion in terms of effective interventions, policies, and strategies to address these diseases of despair. Over the past two decades, mortality from overdose, suicide, and alcoholic liver diseases/cirrhosis has increased across the United States, but the disparity between Appalachia and the non-Appalachian U.S. continues to grow.

## INTRODUCTION

**Figure 1:**
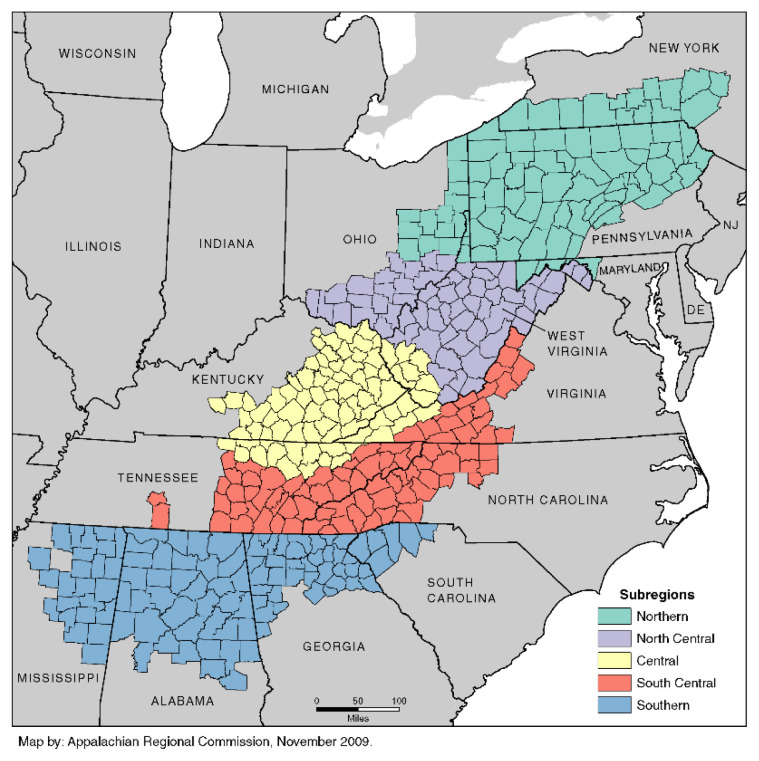
Map of the Appalachian Region

The Appalachian Region is a 205,000-square-mile region that spans from southern New York to northern Mississippi, includes 420 counties and 8 independent cities in 13 states, and has a population of 25 million people (Figure 1).^1^ Appalachia lags behind the rest of the nation in educational attainment, economic development, and health outcomes.^2^,^3^ Appalachia’s median household income is 83% of the U.S. average, and the region’s poverty rate is 16%.^2^,^4^ Certain Appalachian subregions experience greater disparities than others; for example, labor force participation, household income, and bachelor’s degree attainment are lowest in Central Appalachia.^2^,^4^–^6^ A number of studies have shown that the residents of the Appalachian Region experience significant health disparities compared to the rest of the nation.^3^,^7^–^10^

Research conducted by Case and Deaton has demonstrated increasing morbidity and mortality from three main causes—alcohol, prescription drug and illegal drug overdose; suicide; and alcoholic liver disease/cirrhosis of the liver—which have been referred to collectively as “diseases of despair.”^11^ The rise in overdose mortality described by Case and Deaton was driven by the opioid crisis in the U.S. According to data from the Centers for Disease Control and Prevention (CDC), in 2017, the number of overdose deaths involving opioids was six times higher than in 1999.^12^ Socioeconomic factors, including education, employment, and income, are potential factors influencing the growing opioid crisis in the U.S. This study investigated mortality due to diseases of despair within the Appalachian Region, with an additional focus on deaths attributable to opioid overdose.

## METHODS

This study aimed to detect differences in the mortality rates from diseases of despair between the Appalachian Region and the non-Appalachian U.S. (the rest of the country, excluding Appalachia), in addition to differences by age groups and gender. Appalachian rates were further analyzed by subregion, county economic status, and levels of rurality. Appalachian subregions, as defined by the Appalachian Regional Commission (ARC), represent contiguous geographies of relatively homogeneous characteristics (topography, demographics, economics, and transportation) and include: Northern, North Central, Central, South Central, and Southern Appalachia. ARC also provides county-level economic classifications based on an index of three economic indicators (3-year unemployment rate, per capita market income, and poverty rate). Counties are designated based on the index as distressed, at-risk, transitional, competitive, or attainment.^13^ Lastly, ARC designations for rurality were used for these analyses. These designations of large metro, small metro, nonmetro adjacent to large metros, nonmetro counties adjacent to small metros, and rural counties are based on a simplification of the UDSA’s Economic Research Services (ERS) 2013 Urban Influence Codes (UIC).^14^

The majority of the findings presented are from 2015 mortality data from the National Center for Health Statistics (NCHS) National Vital Statistics System (NVSS) Multiple Cause of Death database, accessed through the CDC Wide-ranging Online Data for Epidemiologic Research (CDC WONDER).^15^ Select findings include data through 2017. The Multiple Cause of Death database provides the underlying cause-of-death, as well as up to 20 additional multiple causes, as reported on an individual’s death certificate by a physician, coroner, and/or medical examiner.^16^ Deaths are coded to the International Classification of Disease Tenth Revision (ICD-10) codes.

These analyses included the ICD-10 codes referenced by Case and Deaton, reflecting the underlying cause of death from each of the three diseases of despair: alcohol, prescription drug and illegal drug overdose (X40–X45, Y10–Y15, Y45–Y49); suicide (Y87.0, X60–X84); and alcoholic liver disease/cirrhosis of the liver (K70, K73–K74).^11^ Multiple cause-of-death ICD-10 codes (T40.0, T40.1, T40.2, T40.3, T40.4, T40.6) that specify the type of drug causing the overdose were used to determine the percentage of alcohol, prescription drug and illegal drug overdose deaths attributed to opioids.^17^

The findings present age-adjusted mortality rates for the population aged 15 to 64. Additional analyses provide a more granular focus on mortality rates by age group (10-year increments between ages 15 and 64). Statistical significance was assessed at the 0.05 level using two-sided significance tests (z-tests).

## RESULTS

**Figure 2:**
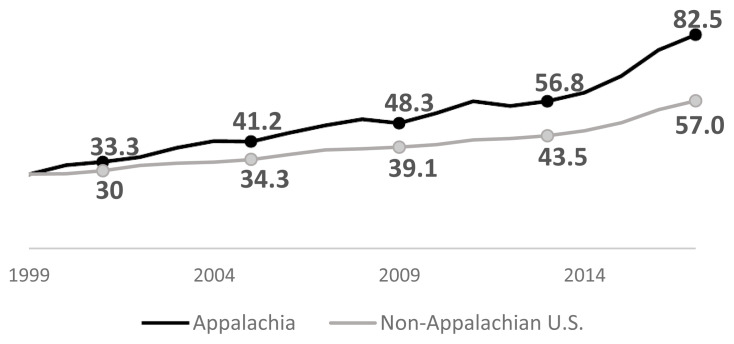
Annual mortality rates attributable to diseases of despair, ages 15–64, by region (1999–2017)*‡ ‡Rates are presented as deaths per 100,000 population. Rates are age adjusted. *In all years except 1999, the Appalachian rate is significantly different from the non-Appalachian U.S. rate, p ≤0.05 Source: Mortality Rates and Standard Errors provided by Centers for Disease Control and Prevention, National Center for Health Statistics. Accessed at http://wonder.cdc.gov/mcd-icd10.html

Over the past 2 decades, the mortality rate due to diseases of despair has been increasing across the U.S. While the non-Appalachian U.S. has seen a rise in these deaths, the gap has widened between the Appalachian Region and the rest of the nation. As shown in Figure 2, the combined diseases of despair mortality rate in Appalachia and the non-Appalachian U.S. were nearly identical in 1999. By 2009, the mortality rate in the Appalachian Region was 24% higher than the non-Appalachian U.S., and by 2017, this difference had grown to 45%. Between 2009 and 2017, the rate in Appalachia has nearly tripled, showing the increasing burden associated with these diseases of despair.

**Figure 3:**
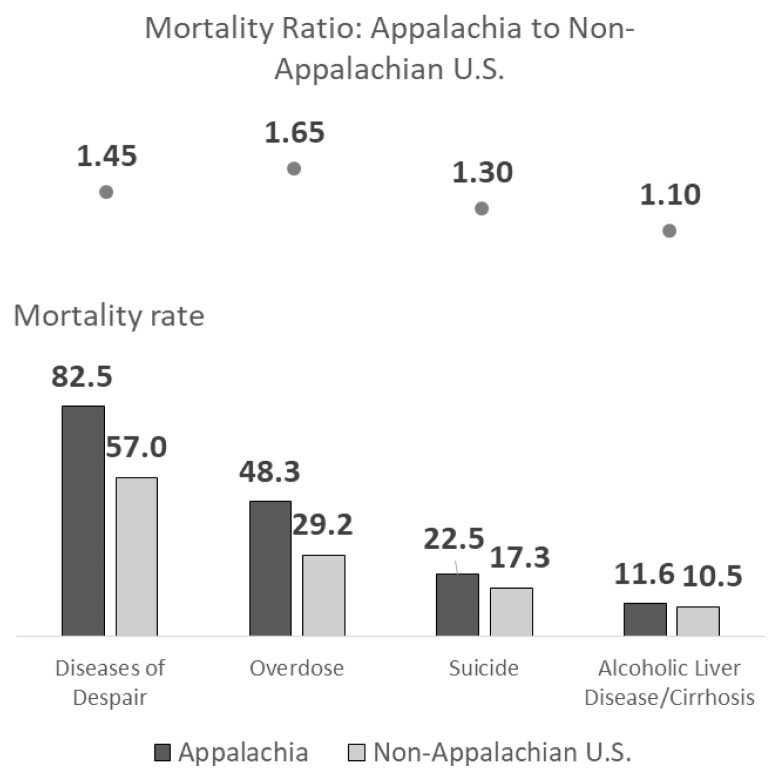
Diseases of despair mortality rates, ages 15–64, by disease and region (2017)*‡ ‡Rates are presented as deaths per 100,000 population. Rates are age adjusted. *For all diseases, the Appalachian rate is significantly different from the non-Appalachian U.S. rate, p ≤0.05 Source: Mortality Rates and Standard Errors provided by Centers for Disease Control and Prevention, National Center for Health Statistics. Accessed at http://wonder.cdc.gov/mcd-icd10.html

As shown in Figure 3, the mortality rate in 2017 for the combined diseases of despair among those aged 15 to 64 years was 82.5 deaths per 100,000 population, compared to 57.0 deaths per 100,000 population for the non-Appalachian U.S. Among the three causes of death (overdose, suicide, and alcoholic liver disease/cirrhosis), the disparity between Appalachia and the non-Appalachian U.S. was greatest for overdose deaths. In 2017, the overdose mortality rate was 65% higher in Appalachia than in the non-Appalachian U.S. (48.3 deaths per 100,000 population in Appalachia compared to 29.2 deaths per 100,000 population in the rest of the U.S.) The suicide mortality rate was 30% higher in Appalachia, and the alcoholic liver disease/cirrhosis mortality rate was 10% higher.

The remaining findings are based on 2015 mortality data. As shown in Table 1, within Appalachia, males were more likely than females to die from overdose, suicide, and alcoholic liver disease/cirrhosis. Among males, the mortality rate for the diseases of despair was 92.7 deaths per 100,000, compared to 41.8 deaths per 100,000 for females. Within Appalachia, individuals aged 45 to 54 had the highest mortality rate from the combined diseases of despair in 2015, at 89.0 deaths per 100,000 population.

Certain subregions of Appalachia face a notable disparity in mortality from overdose, suicide, and alcoholic liver disease/cirrhosis. The mortality rates due to the combined diseases of despair were highest in Central and North Central Appalachia, with rates of 94.4 deaths per 100,000 population and 88.6 deaths per 100,000, respectively. In comparison, the mortality rates in Northern, South Central, and Southern Appalachia were 69.1, 65.1, and 52.3 deaths per 100,000, respectively.

Mortality rates also differed by county economic status. For the combined diseases of despair, the mortality rate was highest for distressed counties, at 83.6 deaths per 100,000 population, compared to 47.2 deaths per 100,000 population for attainment counties. Competitive counties had the second highest mortality rate for the combined diseases of despair (73.1 deaths per 100,000 population.)

For the combined diseases of despair, rural counties had the highest mortality rate at 70.5 deaths per 100,000, compared to 63.2 deaths per 100,000 for large metro counties.

### Overdose Deaths

Overdose deaths have become increasingly common over the past decade, largely due to the opioid crisis, which has greatly impacted Appalachia. In the Appalachian Region in 2015, there were 5594 overdose deaths among those aged 15 to 64 years, and of these, 69% were caused by opioids (opium, heroin, methadone, other opioids and synthetic narcotics). Overdose mortality was 34% higher in economically distressed counties than economically nondistressed (attainment, competitive, transitional, and at-risk) counties. When comparing metro (large metro and small metro) and nonmetro (nonmetro, adjacent large metro; nonmetro, adjacent small metro; and rural) counties, the difference was less notable—the overdose mortality rate in metro counties was 6% higher than the rate in nonmetro counties.

Overdose mortality significantly impacts Appalachian adults between the ages of 25 and 54. Among males, the burden in Appalachia was the greatest among those aged 25 to 34 years (61.9 deaths per 100,000 population) and 35 to 44 years (61.0 deaths per 100,000 population). These age groups also experienced the highest mortality rates in the non-Appalachian U.S., though large disparities were observed when comparing Appalachia to the non-Appalachian U.S. Specifically, the overdose mortality rate was 78% higher among those ages 35 to 44 years and 72% higher among those aged 25 to 34 years in Appalachia than the non-Appalachian U.S. While the overall burden was lower among females than males, the disparity between Appalachia and the non-Appalachian U.S. was greater for females than for males. Among females, the burden in Appalachia was the greatest among those aged 45 to 54 years (35.8 deaths per 100,000 population) and 35 to 44 years (34.7 deaths per 100,000 population). The overdose mortality rate for Appalachian females ages 35 to 44 was more than double the rate for females in the non-Appalachian U.S., and among those aged 25 to 34 years, the Appalachian rate was 92% higher.

As shown in Table 2, states within Appalachia differ in terms of the burden of overdose mortality, and also in the percentage of overdose deaths that were opioid-related. West Virginia had both the highest overdose mortality rate (59.7 deaths per 100,000) and the largest percentage of deaths attributed to opioids, at 88%. In contrast, Appalachian Mississippi had an overdose mortality rate of 12.9 deaths per 100,000, of which only 34% were caused by opioids. The greatest burden from overdose is located within the states of Central and North Central Appalachia, specifically, West Virginia, Appalachian Kentucky, and Appalachian Ohio.

## LIMITATIONS

There are several limitations to this study. First, while we highlight the significant increase in overdose deaths between 2015 and 2017, the analyses presented for subgroups, including age, gender, economic status, and rurality, are based on 2015 mortality data. Due to the rapidly evolving nature of the opioid crisis in the U.S., some of these differences have likely changed since 2015. Secondly, because we conducted analyses for the diseases of despair individually, suicides that are attributable to opioids are not included in the overdose category. Finally, the data on opioid-related overdoses are based on death certificate reporting, and there are known variations by state in terms of the quality of this data.

## DISCUSSION

These findings document the scale and scope of the problem in Appalachia and highlight the need for additional research and discussion in terms of effective interventions, policies, and strategies to address these diseases of despair. Over the past two decades, mortality from overdose, suicide, and alcoholic liver diseases/cirrhosis has increased across the U.S., but the disparity between Appalachia and the non-Appalachian U.S. continues to grow. Within Appalachia, the burden is concentrated within the Central and North Central subregions, where the majority of economically distressed counties are located. Economic development strategies and interventions that address other underlying contributors to the diseases of despair, in addition to increased access to treatment services, prevention, and overdose medications, may be important considerations in addressing this problem.

The rise in opioid overdoses over the past several years has contributed to some of the recent increases in diseases of despair. The states with the highest mortality from overdose in the Appalachian Region are the ones most significantly impacted by the opioid crisis, as shown by the large percentage of overdose deaths attributed to opioids. When comparing Appalachia to the non-Appalachian U.S., the most notable disparities in overdose deaths existed for the group aged 25–44 years. Young adults in Appalachia are considerably more likely to die from an overdose than similar-aged adults in the rest of the U.S., which has significant implications, particularly in terms of economic development, as individuals in their prime working years are most impacted.

The 2017 data presented shows the rapid increase in overdose deaths due to the more powerful synthetic opioids, such as fentanyl; however, these rates have likely continued to rise, and the burden within the Appalachian Region is likely greater than described in these findings. It is critical to continue to track trends in diseases of despair, and particularly opioid-related mortality, to assess the growing burden related to fentanyl and other synthetic opioids.

**SUMMARY BOX**

**What is already known about this topic?** Work conducted by Case and Deaton (2015) demonstrated disparities in mortality resulting from overdose, suicide, and alcoholic liver disease among Caucasian working age adults, collectively termed diseases or deaths of despair.

**What is added by this report?** The work described in this paper expands upon that of Case and Deaton by providing a focused exploration of diseases of despair within the Appalachian region. Findings confirm those of Case and Deaton and provide evidence of disparities focused most prominently within the Central and North Central Appalachian subregions.

**What are the implications for public health practice, policy, and research?** Beyond direct implications related to greater mortality burden within the population of working age adults in the Central and North Central Appalachian subregions, diseases of despair have cascading impacts on regional economic development, children’s health and well-being, and social support systems, among others. Increasing access to treatment services, harm reduction programs and prevention initiatives will benefit individuals with substance use disorder and provide broader community benefit as rates of substance misuse and overdose decline. More broadly, understanding differences in mortality rates at the subregion and population group levels support resource allocation and response efforts.

## Figures and Tables

**Table 1:** Diseases of Despair mortality rates, ages 15–64 (2015)‡

	Overdose	Suicide	Alcoholic liver disease/cirrhosis	Combined Despair Deaths
**Region**				
Appalachia	35.4	19.8	11.4	66.6
Non-Appalachian U.S.	21.5	16.5	10.6	48.6
**Appalachian Subregion**				
Northern	40.2	19.7	9.3	69.1
North Central	56.3	19.4	11.3	88.6
Central	54.9	23.3	16.2	94.4
South Central	30.7	21.1	13.3	65.1
Southern	22.7	18.5	11.0	52.3
**Economic Status in Appalachia**				
Distressed	46.2	22.1	15.4	83.6
Non-Distressed				
At-Risk	33.1	22.9	15.2	71.2
Transitional	33.7	19.4	10.7	63.7
Competitive	45.8	17.5	9.9	73.1
Attainment	26.4	15.2	--	47.2
**Rurality (within Appalachia)**				
Large Metro	37.2	17.2	8.8	63.2
Small Metro	35.5	19.8	12.3	67.5
Nonmetro, adjacent Large Metro	33.6	21.1	11.9	66.7
Nonmetro, adj Small Metro	32.4	22.4	12.1	66.9
Rural	36.2	21.2	13.2	70.5
**Gender (within Appalachia)**				
Male	45.1	31.1	16.0	92.2
Female	25.6	8.5	7.0	41.1
**Age Group (within Appalachia)**				
15–24	12.3	12.6	--	24.9
25–34	44.5	19.4	1.7	65.7
35–44	47.8	21.9	8.3	78.0
45–54	42.7	24.5	21.8	89.0
55–64	23.2	20.7	34.5	78.4

‡ Rates are presented as deaths per 100,000 population. Rates are age adjusted.

Source: Mortality Rates and Standard Errors provided by Centers for Disease Control and Prevention, National Center for Health Statistics.

Accessed at http://wonder.cdc.gov/mcd-icd10.html

**Table 2:** Overdose and opioid-related overdose mortality rates, ages 15–64, by state^ (2015)‡

	Overdose		Opioid-related Overdose		Percent Opioid-related (%)	
**Alabama**	24.7	*	10.8	*	44	
**Georgia**	19.8		14.4		73	
**Kentucky**	52.1	*	38.5	*	74	
**Maryland**	48.4	*	41.4	*	86	
**Mississippi**	12.9	*	4.4	†	34	
**New York**	24.3		17.2	*	71	
**North Carolina**	31.7	*	25.2	*	79	
**Ohio**	49.1	*	40.5	*	82	
**Pennsylvania**	42.0	*	24.7	*	59	
**South Carolina**	30.8	*	22.4	*	73	
**Tennessee**	33.9	*	24.0	*	71	
**Virginia**	26.1		19.6	*	75	
**West Virginia**	59.7	*	52.8	*	88	
**Appalachia**	35.4	*	24.6	*	69	
**Non-Appalachian U.S**.	21.5		16.5		64	

^ For states within Appalachia, only the mortality rate for the Appalachian counties is shown.

‡ Rates are presented as deaths per 100,000 population. Rates are age adjusted.

* Mortality rate is significantly different than the non-Appalachian U.S. rate for the same overdose category, p ≤0.05

† Due to small number of deaths, opioid mortality rate is unreliable and not age adjusted.

Source: Mortality Rates and Standard Errors provided by Centers for Disease Control and Prevention, National Center for Health Statistics.

Accessed at http://wonder.cdc.gov/mcd-icd10.html
